# Are the Self-Employed Mentally Healthier Than Salaried Workers? Evidence From Korea, Mexico, and the United States

**DOI:** 10.1093/geroni/igaa057.132

**Published:** 2020-12-16

**Authors:** Hankyung Jun

**Affiliations:** University of Southern California, Los Angeles, California, United States

## Abstract

Self-employed workers are often reported to have better health than salaried workers. Whether this is because self-employment has health benefits or healthier workers are self-employed is not clear. Self-employed workers may have higher job satisfaction due to higher levels of self-efficacy and autonomy, but may also experience higher job stress, uncertainty, and lack of health insurance leading to mental health problems. Self-employed workers in the U.S. may have different characteristics than those in Mexico and Korea given different working and living environments as well as different institutional arrangements. This study will examine the association between self-employment and mental and cognitive health for older adults in the U.S., Mexico, and South Korea. It uses harmonized panel data from the Health and Retirement Study, the Korean Longitudinal Study of Aging, and the Mexican Health and Aging Study. We compare the health and selection effect of self-employment using a pooled logistic model, fixed-effects model, and a bivariate probit model. In addition to comparing self-employed and salaried workers, we analyze differences between self-employed with and without employees. By using rich data and various models, we address reverse causality and estimate the relationship between self-employment and health. We show that the positive health effects of self-employed workers in the U.S. disappear once controlled for unobserved heterogeneity, indicating the possibility of healthier workers selecting into self-employment. Interestingly, for Korea and Mexico, healthier individuals seem to select into wage work which reflects the difference in working conditions across countries. Further analysis will show effects by business size.

